# Timing of *Fasciola hepatica* infection selectively impairs antibody avidity after foot-and-mouth disease vaccination in cattle

**DOI:** 10.3389/fimmu.2026.1874141

**Published:** 2026-08-11

**Authors:** Conrado Rodríguez, Pablo Parodi, Alejo Menchaca, Anderson Saravia, Maximiliano Wilda, Alejandra Victoria Capozzo, Teresa Freire

**Affiliations:** 1 Laboratorio de Inmunomodulación y Vacunas, Unidad Académica de Inmunobiología, Facultad de Medicina, UdelaR, Montevideo, Uruguay; 2 Instituto Nacional de Investigación Agropecuaria (INIA), Plataforma de Investigación en Salud Animal, Tacuarembó, Uruguay; 3 Centro de Altos Estudios en Ciencias Humanas y de la Salud (CAECIHS). Consejo Nacional de Investigaciones Científicas y Técnicas (CONICET)- Universidad Abierta Interamericana, Buenos Aires, Argentina

**Keywords:** bovine, *Fasciola hepatica*, foot-and-mouth disease, triclabendazole, vaccine

## Abstract

**Introduction:**

*Fasciola hepatica* is a trematode parasite of ruminants that induces potent immunoregulatory responses, potentially compromising vaccine-induced immunity. This study evaluated the effect of *F. hepatica* infection and its timing on immune responses elicited by foot-and-mouth disease virus (FMDV) vaccination in cattle.

**Methods:**

Fifty-two one-year-old calves free of maternal FMDV antibodies were allocated to five experimental groups (10–11 animals/group). Animals were experimentally infected with *F. hepatica* either before or after FMDV vaccination and compared with antiparasitic-treated and non-infected control groups. Growth performance, parasitological parameters, hematological and biochemical indicators of liver function, and vaccine-induced humoral and cellular immune responses were evaluated throughout the study.

**Results:**

Although total anti-FMDV antibody titers and IgG1 levels were not significantly affected by *F. hepatica* infection, calves infected after vaccination developed significantly lower IgG1 antibody avidity indices, indicating impaired antibody maturation. This reduction was accompanied by diminished vaccine-induced cellular immune responses and persistent biochemical evidence of hepatic dysfunction during chronic infection. Triclabendazole treatment restored antibody avidity and partially recovered productive performance, although some metabolic alterations persisted.

**Conclusions:**

*F. hepatica* selectively impaired the qualitative maturation of the immune response to FMDV vaccination rather than the magnitude of antibody production. These findings highlight the importance of infection timing and effective parasite control to optimize vaccine efficacy in cattle and suggest that antibody avidity may represent a more sensitive indicator of helminth-induced vaccine interference than antibody titers alone.

## 1 Introduction

Foot-and-mouth disease (FMD) is a highly contagious vesicular viral disease affecting cloven-hoofed animals, characterized by high morbidity in adult livestock and potentially high mortality in young animals. The circulation of foot-and-mouth disease virus (FMDV) in susceptible populations leads to severe restrictions on animal movement and international trade, resulting in substantial economic losses worldwide. Consequently, vaccination remains the most relevant tool for FMD control in endemic areas of Asia, Africa, and South America, and an essential component of emergency preparedness in FMD-free nations susceptible to viral incursion (1, 2). Experimental challenge studies in cattle have demonstrated a strong correlation between vaccine-induced antibody titers, commonly measured by liquid-phase blocking ELISA (LPBE) or the virus neutralization test, and protection against clinical disease. In South America these correlations were validated for LPBE titers measured at 60 days post-vaccination (60 dpv) and challenge at 90 dpv (3). These correlations enable the prediction of protective immunity without the need for live virus challenge, aligning with animal welfare principles and the 3R framework (Replacement, Reduction, and Refinement) (2, 4). Beyond antibody magnitude, increasing evidence indicates that qualitative features of the humoral response, including antibody avidity (5), affinity maturation, IgG isotype distribution (6) and IgG1 avidity indexes (7), play a critical role in determining the breadth, durability, cross-protection and field efficacy of vaccine-induced protection against FMDV (2, 8).

Bovine fasciolosis, caused primarily by *Fasciola hepatica* and *Fasciola gigantica*, is one of the most prevalent helminth infections of ruminants worldwide and represents a major constraint to livestock productivity. Global economic losses attributed to fasciolosis exceed USD 3 billion annually, largely due to reduced weight gain, impaired feed efficiency, decreased milk production, reduced fertility, liver condemnation at slaughter, and increased susceptibility to secondary infections (9, 10). In cattle, pathology arises from the migration of immature flukes through the hepatic parenchyma during the acute phase of infection, followed by chronic bile duct inflammation and fibrosis once adult parasites establish in the biliary system (11). Triclabendazole (TCBZ) is the primary control measure, due to its efficacy against all stages of *F. hepatica*, although its sustainability is now challenged by increasing drug resistance. However, even successful treatment may not fully reverse the hepatic and immunological damage caused by chronic infection, thereby continuing to hinder vaccine effectiveness (9, 12, 13).

Beyond its direct pathological effects, *F. hepatica* acts as a potent immunomodulator by inducing a systemic shift toward a polarized Th2 and regulatory immune environment. This response is characterized by the upregulation of IL-4, IL-10, and TGF-β, the expansion of regulatory T cells (Tregs), and the differentiation of alternatively activated (M2) macrophages and tolerogenic dendritic cells (14). While these mechanisms likely promote parasite survival and mitigate host tissue damage, they simultaneously create a state of “bystander” immunosuppression that impairs responses to unrelated antigens and reduces vaccine efficacy (15). Some studies have shown that helminth infections can interfere with vaccine-induced immunity. In cattle, earlier work evaluating immune responses to bacterial or respiratory vaccines during *F. hepatica* infection reported limited effects on total antibody titers (16, 17). However, more recent studies suggest that fasciolosis can selectively impair qualitative aspects of immune responses, including antibody avidity and cell-mediated immunity, without necessarily reducing overall antibody levels (9).

Given the immunoregulatory nature of *F. hepatica* and the global importance of FMD vaccination, understanding the interaction between liver fluke infection and vaccine efficacy is paramount. This study comprehensively evaluated the impact of *F. hepatica* on bovine productive performance, liver function, and the quantitative and qualitative immune response to FMD vaccination in two different settings, with vaccination either before or after parasite infection. Furthermore, we assessed how TCBZ treatment and the timing of infection influence antibody maturation and long-term functional immunity in controlled conditions.

## 2 Materials and methods

### 2.1 Parasite experimental infection

Fifty-two 12-month-old male calves (40 Braford and 12 Hereford) were used in this study. The mean body weight for animals was: group i) 164.3 ± 17.0 Kg; group ii) 166.1 ± 17.5 Kg, group v) 172.0 ± 22.2 Kg, group iii) 200.8 ± 26.0 Kg and group iv) 201.6 ± 18.4 Kg. All animals were confirmed negative for *F. hepatica* via two consecutive sedimentation tests (18) prior to experimental infection. Upon arrival, animals were treated with Ricoverm (Konig; 1 mL/40 kg body weight) and remained free of gastrointestinal parasites, as confirmed by the modified McMaster technique (19), until the experimental *F. hepatica* infection. The study was conducted at the La Magnolia Experimental Station (INIA Tacuarembó, Uruguay), located approximately 15 km east of Tacuarembó city (31°42′32″ S, 55°49′36″ W), at an altitude of 115 m above sea level. The region has a humid subtropical climate with no marked dry season, characterized by a mean annual temperature of approximately 18 °C and an average annual rainfall of approximately 1,050 mm. The experimental station was free of intermediate snail hosts, preventing natural *F. hepatica* transmission during the study period. A map of the study area is provided in **Supplementary Figure 1** The calves were kept outdoors under pasture-based conditions on natural grassland and supplemented with commercial feed at 1% of body weight with *ad libitum* access to water throughout the study.

**Figure 1:**
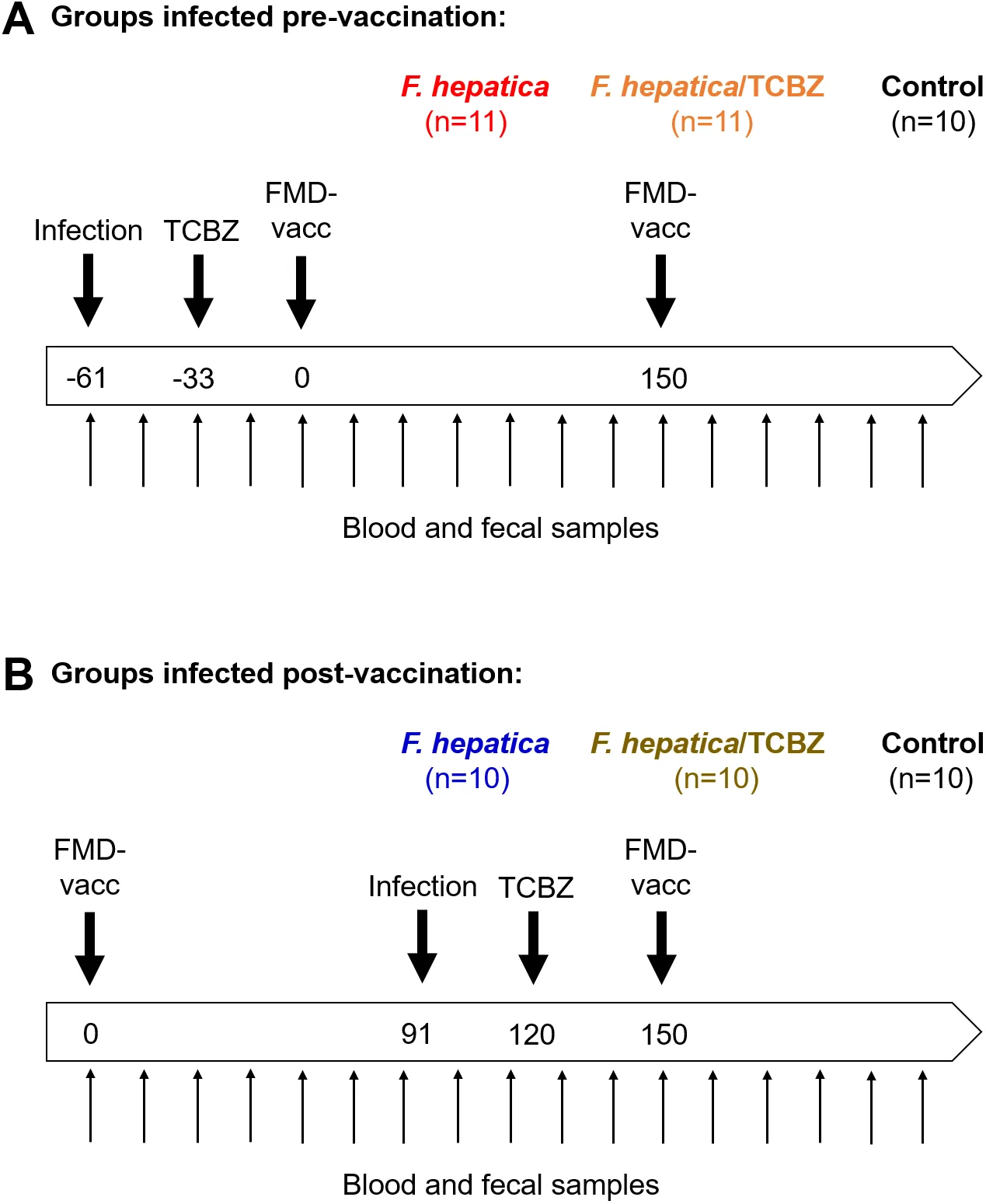
Timeline of parasitic infections and treatments in the different animal groups. **(A)** Pre-vaccination infection. **(B)** Post-vaccination infection. The control group was the same for all the animal groups (total n=52).

Animals were divided into five groups of (n=10–11 per group) balanced by age, weight and breed: i) infected pre-vaccination, ii) infected pre-vaccination and TCBZ-treated, iii) infected post-vaccination, ii) infected post-vaccination and TCBZ-treated, and v) control groups (**Figure 1**). Groups i) and ii) were infected with *F. hepatica* prior to FMD vaccination (**Figure 1A**), while groups iii) and iv) were infected after vaccination (**Figure 1B**). Experimental infections were carried out with 2-2.5 TCBZ-sensitive metacercariae (Ridgeway Laboratories, England) per animal kg, spread in saline solution, inserted into gelatine capsules (Torpac™) and delivered orally using a dosing gun. As control, non-infected calves (n=11) were maintained under the same conditions of infected animals during the experiment. At 30 days post-infection (dpi), animals in groups ii) and iv) received a subcutaneous dose of TCBZ (Triclamax, Grappiolo y Cia. S.A.) at 1 mL/30 Kg as per the manufacturer’s instructions. All procedures involving animals were approved by INIA’s Committee on Animal Research (CNEA Protocol Number: 0009/11).

### 2.2 FMD vaccination

Calves were vaccinated twice at day 0 and day 149 against FMDV with the full dose (2 mL) of the commercially available inactivated oil-adjuvant FMD vaccine Bioaftogen bivalent vaccine from Biogénesis Bagó (series 1097, Buenos Aires, Argentina) formulated with A24/Cruzeiro and O1/Campos strains administered intramuscularly. The vaccination was performed in the context of the compulsory national program of vaccination against FMD in Uruguay.

### 2.3 Collection of samples

Animals were weighted and faecal and blood samples were collected every two to four weeks. at days 0 (1^st^ vaccine dose), 14, 28, 63, 91, 105, 119, 149 (2^nd^ vaccine dose), 163, 177, 191, 205, 219. Additionally, samples from animals from groups i), ii) and v) were collected on days -61, -42, -28 and -14 (**Figure 1**). Blood was collected in tubes containing anticoagulant (EDTA), which were centrifuged after the complete blood count was performed to obtain plasma. The fecal egg count (FEC) of *F. hepatica* was determined individually using the sedimentation technique (18).

### 2.4 Hemogram and circulating leukocyte counting

Blood samples were processed using an automated hematology analyzer (BC-5000 vet; Mindray, China). Total white blood cell (WBC) counts, including differentiation of neutrophils, lymphocytes, monocytes, eosinophils, and basophils, erythrocyte count, hematocrit, mean corpuscular volume (MCV), mean platelet volume (MPV), and platelet counts were analyzed.

### 2.5 Hepatic synthetic functions and transaminase determination

Plasma levels of albumin (ALB; g/dL), total protein (TP; g/dL), aspartate aminotransferase (AST; IU/L), alanine aminotransferase (ALT; IU/L), gamma glutamyltransferase (GGT; IU/L) and total bilirubin (TBil; mg/dL) were determined using an automated spectrophotometer (Dimension^®^ RxL Max Auto-Analyzer; Siemens Healthcare Diagnostics Ltd).

### 2.6 Evaluation of *F. hepatica*- specific antibodies

Parasite specific antibodies were detected by an in-house ELISA using protein lysates from adult parasites as described previously (8). Briefly, adult worms obtained from the bile ducts of infected bovine livers, were mechanically disrupted and sonicated. After centrifugation at 40,000 × g for 60 min, supernatants were collected and dialyzed against PBS. The obtained lysate (FhTE) was resuspended on PBS containing a cocktail of protein inhibitors (Sigma-Aldrich, St. Louis, MO) and dialyzed against PBS for 24 h, before determining protein concentration by bicinchoninic acid assay (Sigma-Aldrich, St. Louis, MO). FhTE (1 μg/well) in 50 mM carbonate buffer (pH 9.6) was coated overnight at 4 °C in ninety-six-well microtiter plates (Nunc, Roskilde, Denmark). After blocking with 1% gelatin in PBS, three washes with PBS containing 0.1% Tween-20 were performed. Serially diluted sera in buffer (PBS containing 0.1% Tween-20 and 0.5% gelatin) were added to the wells for 1 h at 37 °C. Following three washes, wells were treated for 1 h at 37 °C using sheep anti-bovine IgG peroxidase-conjugate (Biorad, CA) and o-phenylenediamine-H_2_O_2_ was added as substrate. Plates were read photometrically at 492 nm in an ELISA spectrophotometer (Labsystems Multiskan MS, Finland). Serum samples were run in two-fold serial dilutions starting at 1:100 and antibody titers were expressed as arbitrary units (AU) using the same calibrator in every plate.

### 2.7 FMDV-specific IgG subtypes

IgG isotypes levels against A24/Cruzeiro were detected by indirect ELISAs as reported by Lavoria et al. (5), using 146S purified viral particles as capture antigen. Plates were revealed using anti-IgG1 peroxidase conjugate antibodies (Biorad, CA) and 3,3′,5,5′-Tetramethylbenzidine (TMB) as chromogen. Serum samples were run in two-fold serial dilutions starting at 1:50 and isotype antibody titers were expressed as the highest dilution of the serum reaching an optical density (OD) equal to the mean OD obtained from negative sera ± 2 standard deviations (SD).

### 2.8 Avidity of FMDV-specific antibodies

FMDV-specific total IgG and IgG avidity were determined using a single dilution indirect ELISA performed as described previously (5). Briefly, plates were coated with 146S sucrose-gradient purified particles (A24/Cruzeiro), blocked, washed and serum samples (diluted 1:50) were run in duplicates. One of the wells was washed with PBS and the other with PBS containing 6 M Urea to detach low-avidity binders. The presence of specific antibodies was revealed using peroxidase-conjugates against bovine total IgG and IgG1 (Biorad, CA, as per 7). OD values for samples and controls were corrected by subtracting mean blank OD values. The avidity index was estimated by the ratio between Urea and PBS treated samples (cOD) multiplied by 100.

### 2.9 Antibodies against non-structural (NS) protein

Antibodies against the highly conserved 3B non-structural protein were determined using a commercial kit following the manufacturer’s instructions (PrioCHECK™ FMDV NS Antibody ELISA Kit, ThermoFisher Scientific).

### 2.10 Liquid-phase blocking ELISA

Total anti-FMDV A/24 Cruzeiro antibody responses were assessed by LPB-ELISA performed as stated by the WOAH Manual (20), using a commercial kit (K-FLP-A24, CEVHAN). The kit provides strain specific rabbit antisera to capture inactivated virus and a monoclonal antibody pool as detection antibodies, followed by an anti-mouse peroxidase conjugate (Jackson, USA). Antibody titers were expressed as the reciprocal log10 of serum dilutions giving the 50% of the absorbance recorded in the virus control wells without serum.

### 2.11 IFN-γ production

IFN-γ production was assessed in stimulated plasma collected from whole blood cultures stimulated *ex vivo*. Briefly, heparinized blood samples were cultured in the presence of inactivated FMDV 146S sucrose-gradient purified particles (21), pokeweed mitogen (PWM, positive control) or mock-treated for 72 h at 37 °C in a humidified atmosphere with 5% CO_2_. After incubation, culture supernatants were harvested, and IFN-γ concentrations were quantified using a sandwich ELISA (Innovative Diagnostics, IDvet) according to the manufacturer’s instructions.

### 2.12 Statistical analysis

Results were analysed using GraphPad Prism software 6.0 (GraphPad Software, San Diego, CA) by non-parametric (with Krustal-Wallis test) or parametric one-way ANOVA followed by the Krustall Wallis test for multiple comparisons, according to the experiment. Antibody titers induced after vaccination and measured by LPBE were analyzed to fit the expected percentage of protection (EPP). The EPP estimates the likelihood that cattle would be protected based on LPBE titers measured at 60 dpv and results from the homologous challenge performed at 90 dpv using the PP method on 16 naive cattle. EPP 75% for A24/Cruzeiro LPBE is 1.90 (Log 10 titer). Asterisks represent statistically significant differences as follows: *p *<* 0.05, **p *<* 0.01, ***p *<* 0.001 and ****p *<* 0.0001.

## 3 Results

### 3.1 *F. hepatica* infection reduces cumulative weight gain in vaccinated cattle in a timing-dependent manner

**Figure 2:**
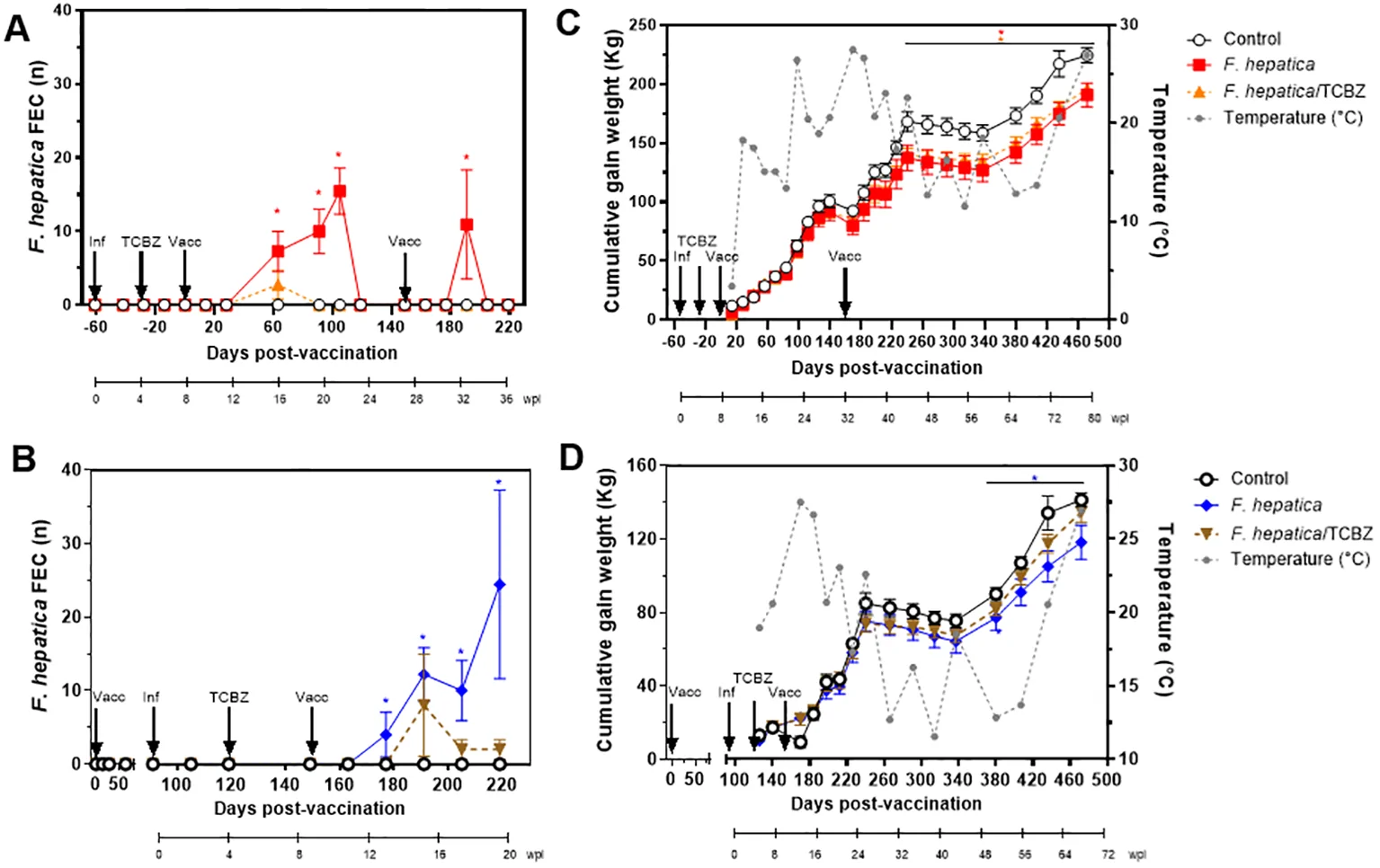
Parasitic burden in infected animals and cumulative weight gain. Eggs per gram (EPG) of feces were determined over a 220-day period using the McMaster sedimentation method. **(A)** Infection prior to vaccination. **(B)** Infection after vaccination. Cumulative weight gain of control and infected animals prior **(C)** or after **(D)** vaccination. Asterisks indicate significant differences compared to the control group (*p* < 0.05). Statistical significance was calculated using one-way ANOVA.

*F. hepatica* infection was evidenced by the presence of eggs in fecal samples approximately 120 dpi (**Figures 2A, B**). Treatment with TCBZ significantly reduced the number of FEC, although no complete elimination of FEC was observed. No significant differences were observed in gastrointestinal nematode infections or *Eimeria* spp. between infected groups and the control group (**Supplementary Figure 2**).

Animals infected prior to vaccination showed a significantly lower total weight gain compared to controls starting at day 220 post-vaccination, an effect that was not reversed by TCBZ treatment (**Figure 2C**). In contrast, when parasitic infection occurred after vaccination, a reduced weight gain was observed in infected animals but not in infected and treated animals, from day 380 post-vaccination onwards (**Figure 2D**). Low ambient temperature levels coincided with a lack of increase of cumulative weight gain.

### 3.2 Parasitic infection induces leukocytosis and sustained eosinophilia without overt anemia

**Figure 3:**
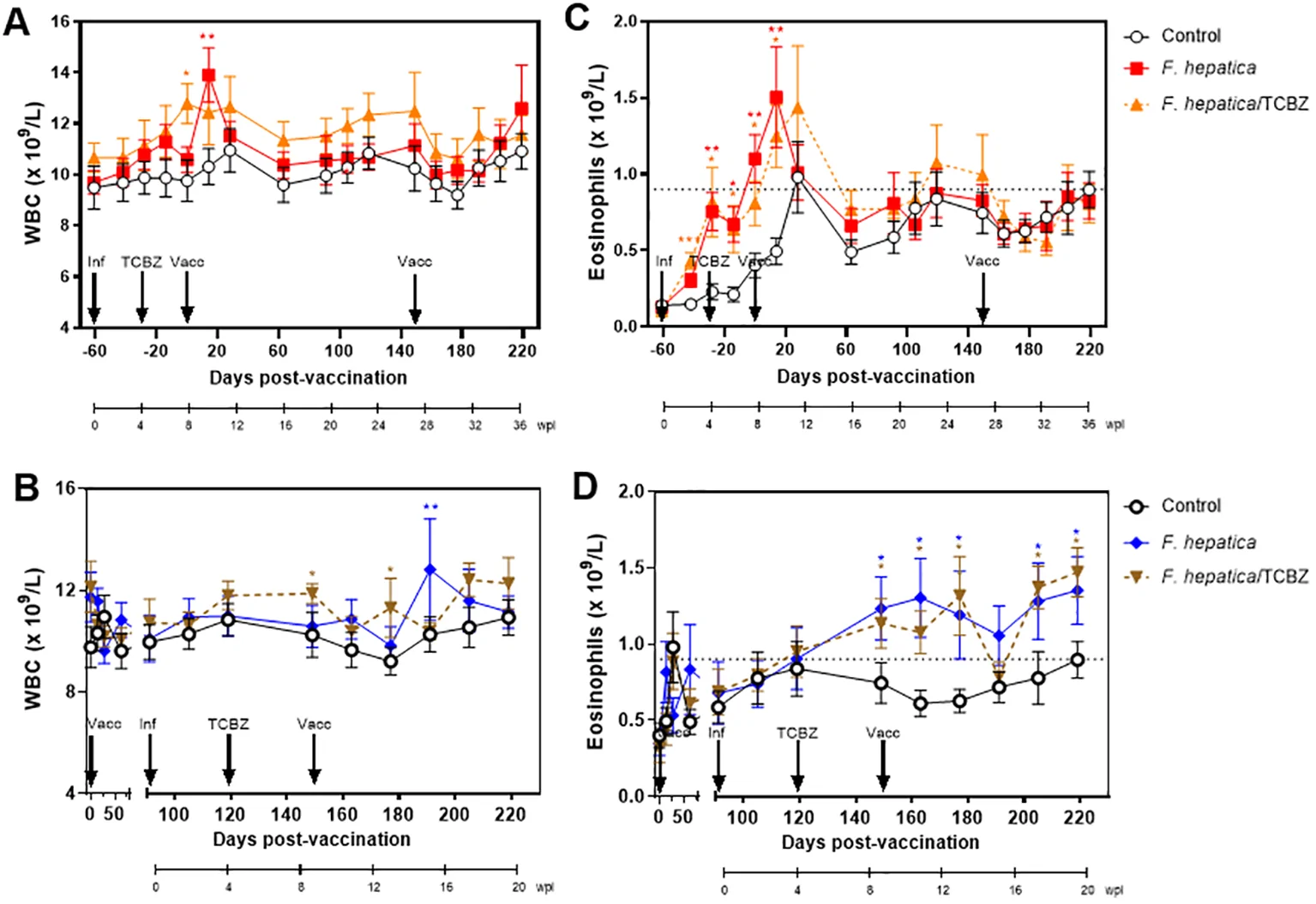
White blood cell and eosinophil counts in infected and control animals. Absolute counts of circulating white blood cells and eosinophils were determined by hemocytometry. **(A,B)** White blood cells. **(C,D)** Eosinophils. Asterisks indicate significant differences compared to the control group (*p* < 0.05; *p* < 0.01). Statistical significance was calculated using one-way ANOVA.

No signs of anemia associated with infection were detected, and no significant changes were observed in platelet counts, red blood cell counts, or other analyzed parameters (**Supplementary Figure 3**). In contrast, moderate leukocytosis and a significant increase in circulating eosinophils were observed from 30–70 dpi onwards in both experimental infection schemes, regardless of antiparasitic treatment (**Figures 3A, B**). This increase is consistent with helminth infection, in which an increase of eosinophils is detected in the acute phase of infection (**Figures 3C, D**).

### 3.3 *F. hepatica* infection causes hepatocellular damage and cholestasis associated with impaired hepatic synthetic capacity independently of the treatment

**Figure 4:**
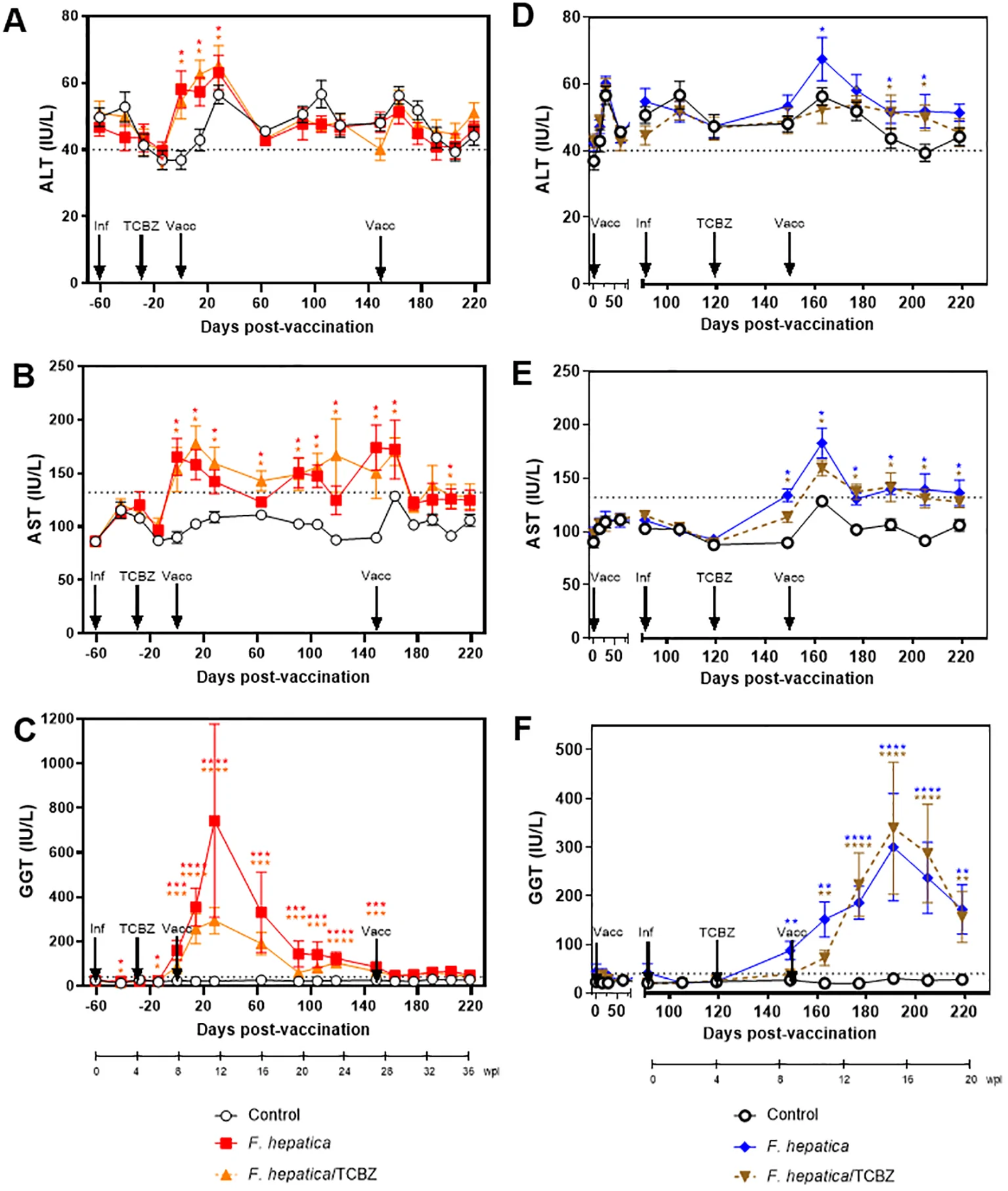
Serum transaminase levels. **(A–C)** Animals infected prior to vaccination. **(D–F)** Animals infected after vaccination. **(A, D)** ALT. **(B, E)** AST. **(C, F)** GGT. Asterisks indicate significant differences compared to the control group (*p* < 0.05; *p* < 0.01; *p* < 0.001; *p* < 0.0001). Statistical significance was calculated using one-way ANOVA.

Modest but significant increases in ALT were detected after 60 dpi. Furthermore, AST activity was significantly higher from 60 dpi onwards in all infected animals (**Figures 4A, B, D, E**), indicating hepatic injury. GGT activity increased later, suggesting the development of chronic cholestasis, with lower values observed in animals infected after vaccination (**Figures 4C, F**). TCBZ treatment did not significantly modify these parameters, except for an attenuation of ALT elevation in animals infected after vaccination (**Figure 4D**).

**Figure 5:**
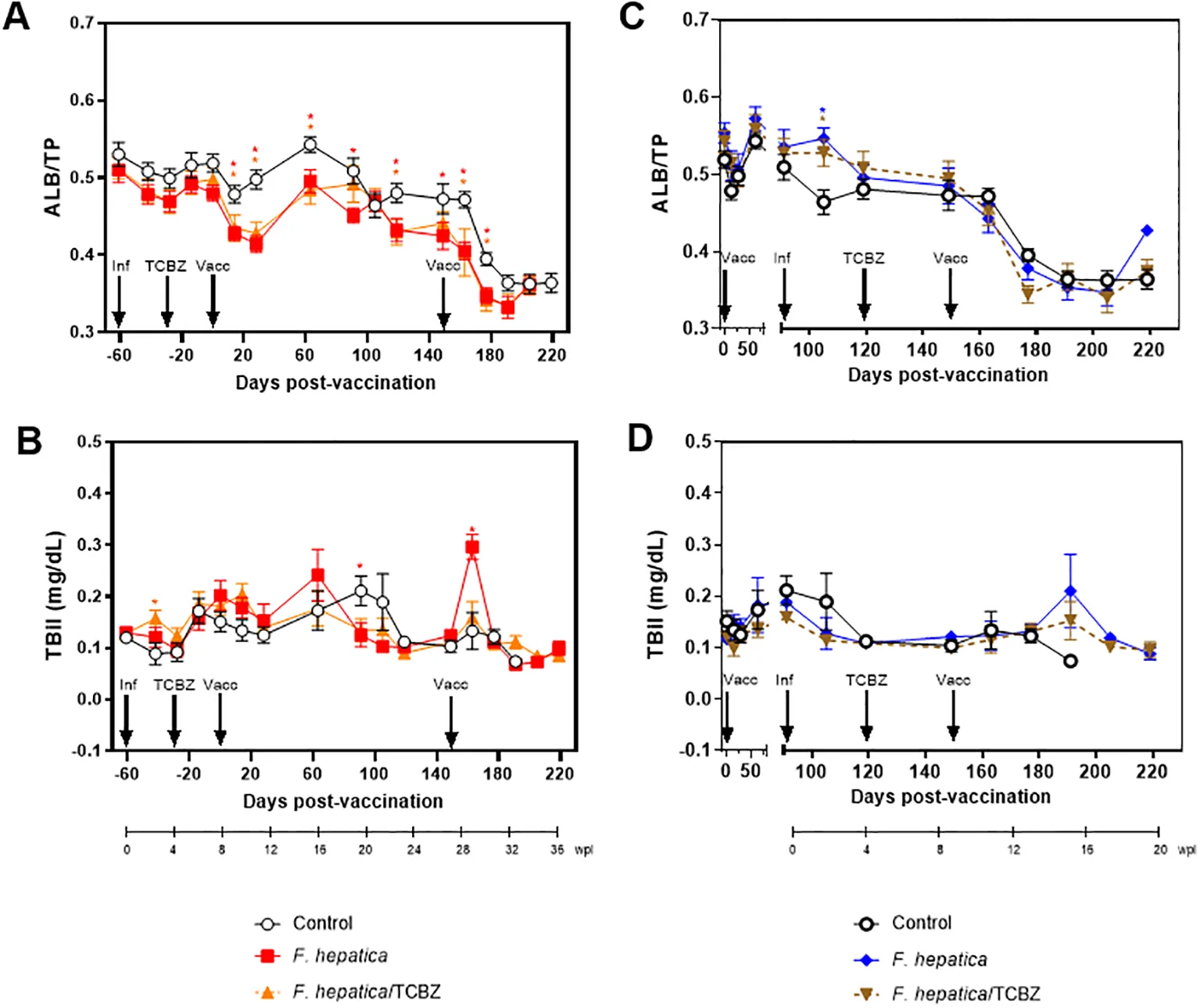
Hepatic capacity for bilirubin and protein synthesis. **(A, B)** Animals infected prior to vaccination. **(C, D)** Animals infected after vaccination. **(A, C)** Albumin-to-total protein ratio. **(B, D)** Total indirect bilirubin. Statistical significance was calculated using one-way ANOVA.

A reduced hepatic protein synthesis capacity, expressed as the albumin-to-total protein ratio, was also detected, with a more pronounced reduction in animals infected prior to vaccination (**Figures 5A, C**). This reduction was observed independently of antiparasitic treatment. Finally, total serum bilirubin levels remained largely unchanged in infected animals compared to controls, except at later time points when control animals exhibited a marked increase in bilirubin levels (**Figures 5B, D**).

### 3.4 *F. hepatica* infection elicits a transient parasite-specific IgG response modulated by antiparasitic treatment

**Figure 6:**
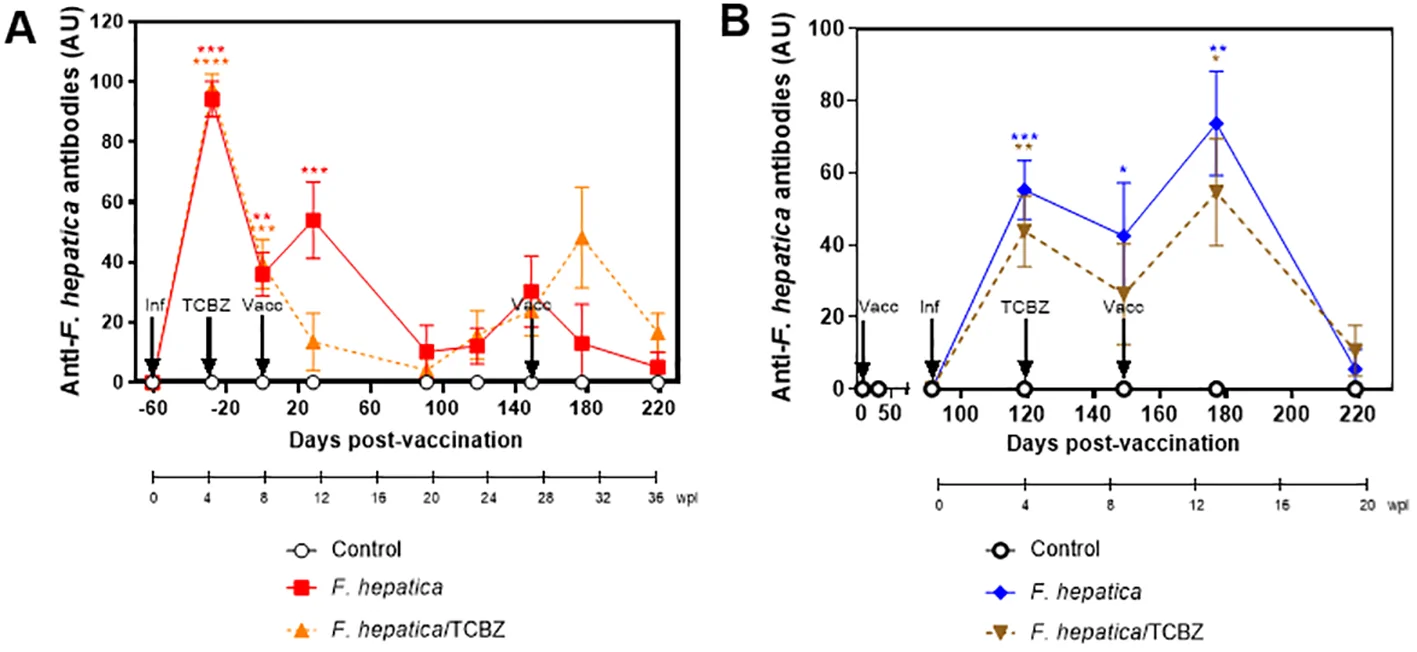
Antiparasitic humoral immune response. Anti-*F. hepatica* IgG antibody levels were quantified by indirect ELISA using a lysate of adult parasite components. A calibrator was used to normalize titers, which were expressed as arbitrary units (AU). **(A)** Infection prior to vaccination. **(B)** Infection after vaccination. Asterisks indicate significant differences compared to the control group (*p* < 0.05; *p* < 0.01; *p* < 0.001; *p* < 0.0001). Statistical significance was calculated using one-way ANOVA.

Parasite-specific antibody response induced by *F. hepatica* infection was evaluated. An increase in anti-*F. hepatica* specific IgG was observed from 30 dpi onwards, followed by a progressive decline after 100–120 dpi. TCBZ treatment reduced the magnitude of the antiparasitic humoral response in both infection schemes (**Figure 6**).

### 3.5 Post-vaccination *F. hepatica* infection selectively affects immune quality rather than antibody magnitude

**Figure 7:**
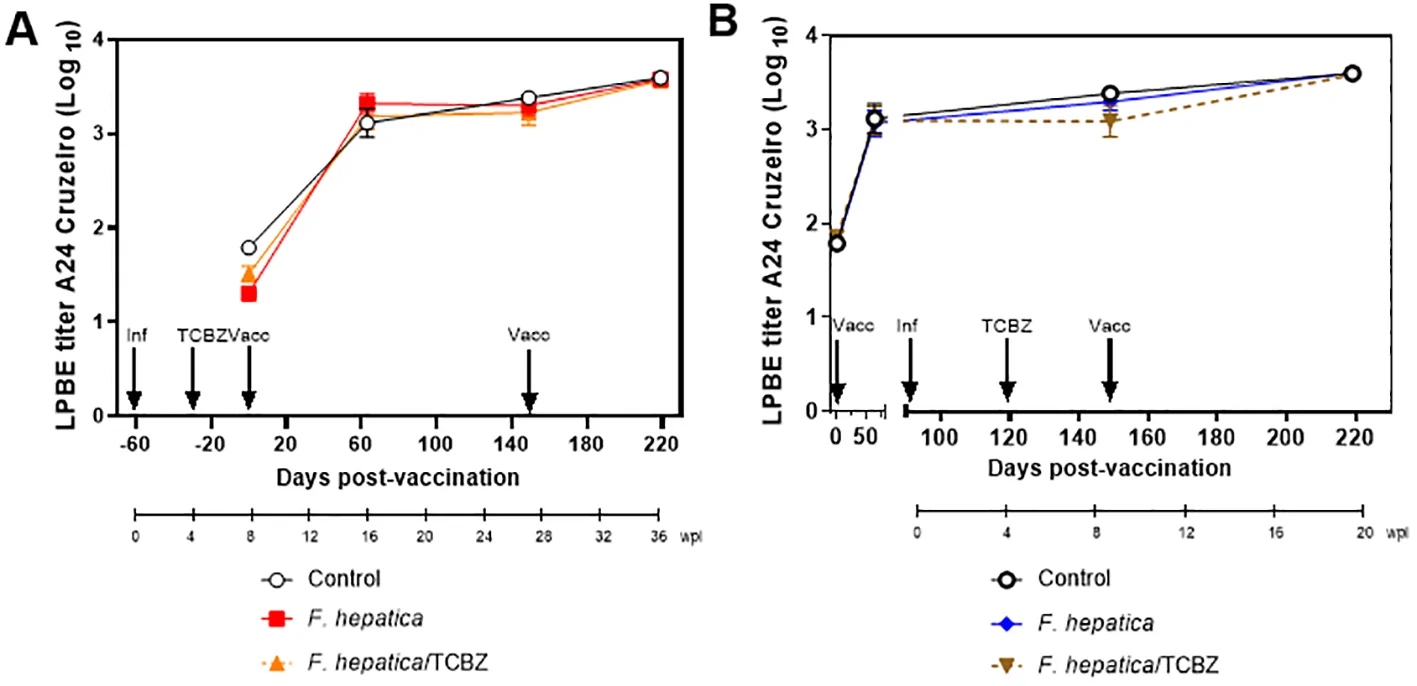
Total antibodies specific to FMDV structural proteins. Antibody levels were quantified by liquid-phase blocking ELISA (LPBE). **(A)** Animals infected prior to vaccination. **(B)** Animals infected after vaccination. No significant differences were detected using one-way ANOVA.

**Figure 8:**
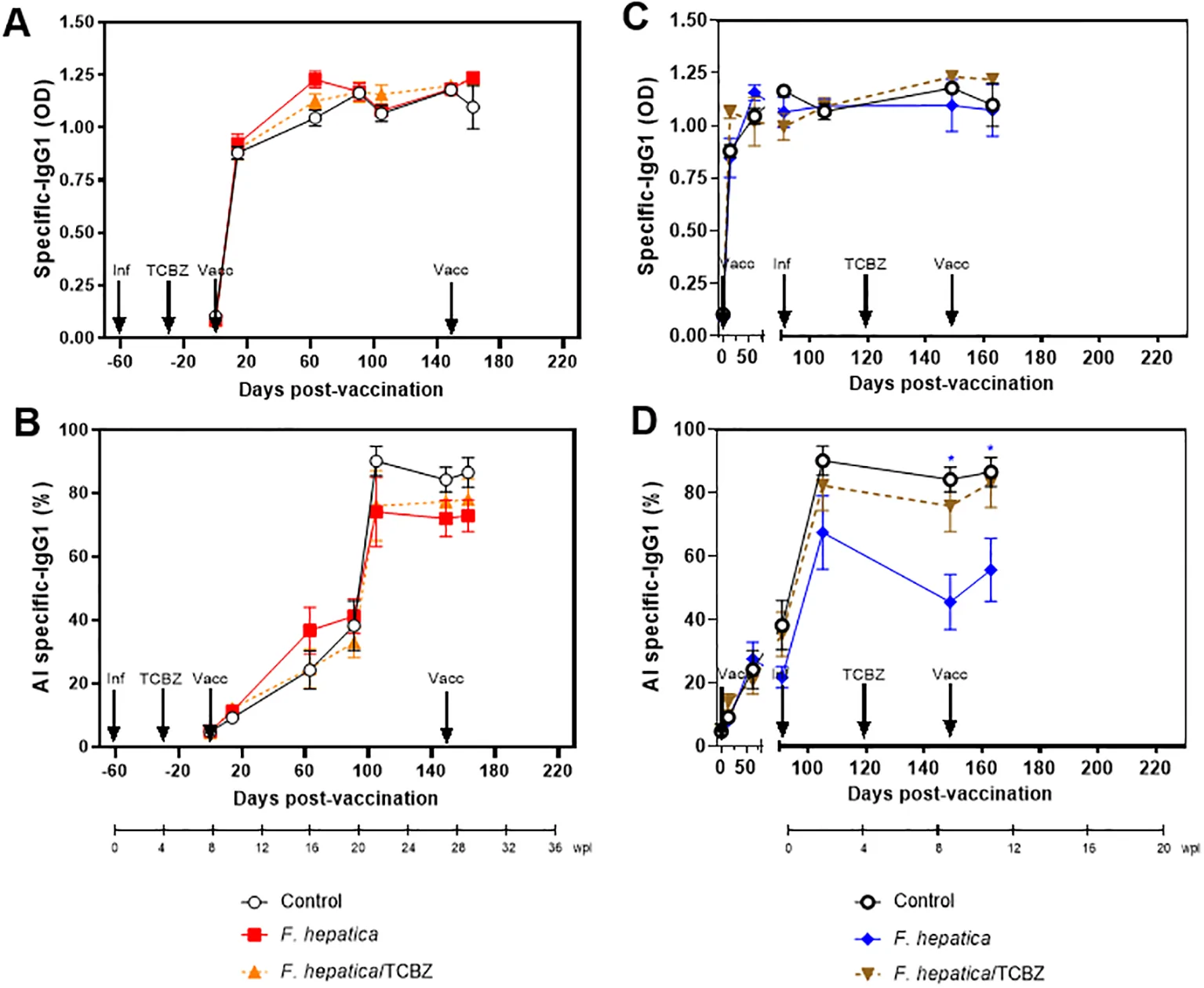
FMDV-specific IgG1 antibodies. **(A, C)** FMDV-specific IgG1 titers quantified by ELISA. **(B, D)** IgG1 avidity index, determined in the presence of 7 M urea. **(A, B)** Animals infected prior to vaccination. **(C, D)** Animals infected after vaccination. Asterisks indicate significant differences compared to the control group (*p* < 0.05). Statistical significance was calculated using one-way ANOVA.

Total antibody titers against FMDV structural proteins were assessed by LPBE and were comparable across experimental groups (**Figure 7**). High titers were already detected after a single vaccination (mean values 16 ±,0.437), exceeding the EPP 75% protective threshold at 60 days post-vaccination (dpv) (log_10_ titer = 1.9 for the A24/Cruzeiro strain). IgG1 antibodies were undetectable prior to vaccination, confirming the absence of maternal antibodies in the vaccinated calves. Vaccination induced robust IgG1 responses in all groups, with similar kinetic profiles regardless of infection status or antiparasitic treatment (**Figures 8A, C**).

Despite comparable antibody titers across groups, antibody quality was differentially affected by *F. hepatica* infection. A significant reduction in IgG1 avidity was observed in animals infected after vaccination (**Figure 8D**), whereas no reduction was detected in animals infected prior to vaccination (**Figure 8B**). This decrease in avidity persisted after booster vaccination and was partially attenuated by antiparasitic treatment. Significant differences in mean avidity indexes were observed between TCBZ-treated and untreated animals at 149 and 163 dpv, corresponding to 29 and 43 days post-TCBZ treatment. All animals remained negative for antibodies against FMDV non-structural proteins when assessed prior to vaccination, at 60 dpv, and at the final sampling point, confirming the absence of virus circulation and ensuring that the measured responses were vaccine-induced.

**Figure 9:**
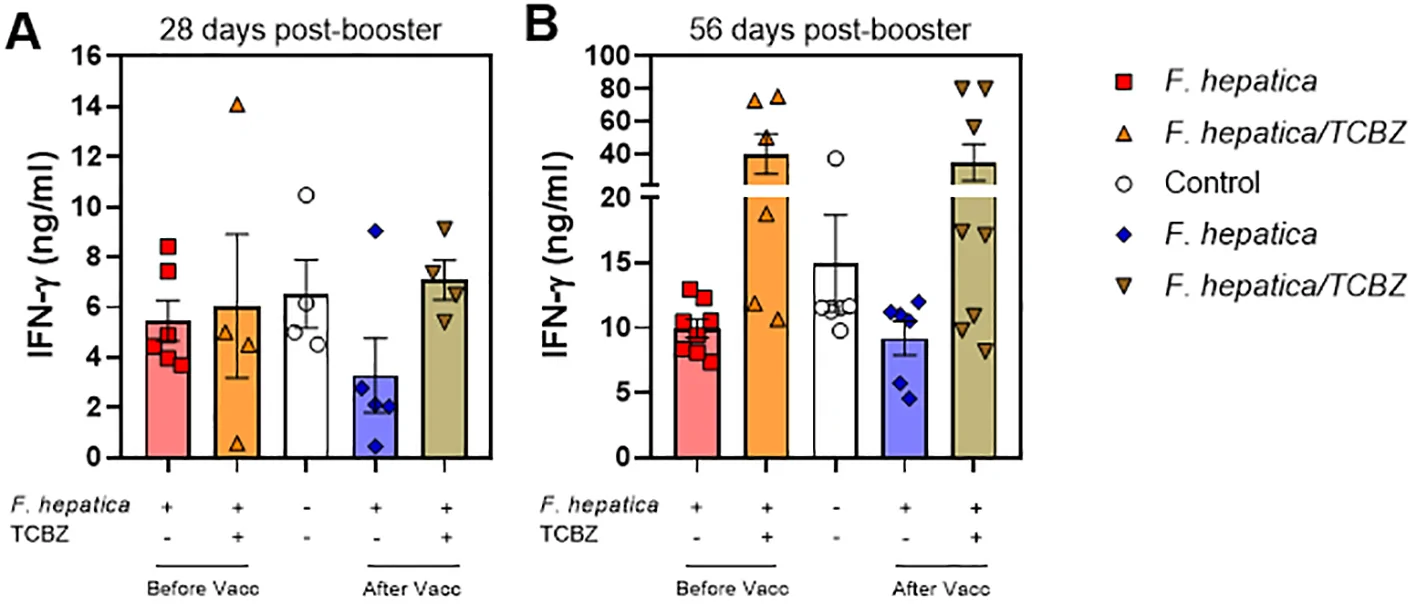
FMDV-specific cellular immune response measured by IFN-γ production. Blood cells were stimulated *ex vivo* with inactivated FMDV, and IFN-γ production was quantified at 191 and 219 dpv, corresponding to 28 **(A)** and 56 **(B)** dpb, respectively.Data are presented as mean ± SD.

In addition to humoral responses, FMDV-specific cellular immunity was assessed by IFN-γ production at 191 and 219 dpv, corresponding to 28 and 56 days post-booster (dpb), respectively. The specific cellular response to FMDV at 28 dpb tended to be reduced in animals infected after vaccination (Ctl vs *F. hepatica-*infected: p=0.1603) and was restored by antiparasitic treatment (*F. hepatica* vs *F. hepatica*/TCBZ p=0.0755) (**Figure 9A**). Similar results were obtained at 56 dpb (**Figure 9B**) with a trend of the *F. hepatica*-infected group after vaccination to decrease against control animals (p=0.1996) and *F.hepatica*/TCBZ group (p=0.0736). In addition, at 56 dpb, TCBZ-treated groups tended to present an increase by IFN-γ production by blood cells with respect to *F. hepatica*-infected animals, although this increase was not significant with respect to control animals (**Figure 9B**).

## 4 Discussion

This study provides evidence that *F. hepatica* infection can differentially impact productive performance, liver function, and vaccine-induced immune responses to FMDV, with effects that depend critically on the timing of parasite infection relative to vaccination. Beyond changes in classical production and biochemical parameters, and while no significant differences were detected in the cellular immune parameters evaluated, our findings reveal that helminth infection selectively compromised qualitative aspects of vaccine-induced humoral immunity, by reducing antibody avidity without altering total antibody levels. Therefore, they highlight an underappreciated mechanism by which endemic parasitic infections can modulate vaccine effectiveness in livestock systems worldwide. It also reveals that overlapping of the early chronic phase of infection with vaccination impairs the avidity of the induced antibodies, and that TCBZ treatment previous to vaccination may prevent these effects.

Experimental infection with *F. hepatica* in both assayed settings resulted in the expected establishment of chronic fasciolosis, as evidenced by egg shedding from approximately 14 weeks post-infection (wpi), and was partially controlled by TCBZ treatment, within the expected variability in intake and outbred animal susceptibility. The lack of significant differences in concomitant gastrointestinal nematodes or *Eimeria* spp. infections supports the interpretation that the observed immunological and physiological effects are primarily attributable to *F. hepatica*. These findings reinforce the suitability of this experimental model to study host–parasite–vaccine interactions under controlled conditions. TCBZ is the drug of choice for the treatment of fasciolosis caused by *F. hepatica*, due to its high efficacy against both immature and adult parasite stages (9, 22). Nevertheless, the increasing prevalence of TCBZ resistance worldwide represents a growing challenge for effective parasite control, leading to persistent infections and prolonged host–parasite interactions (22–24). Consistent with our findings, previous studies have shown that TCBZ may not completely eliminate *F. hepatica* infection, with reported efficacy around 80–90% in terms of FEC reduction under field conditions (25–27).

From a production perspective, animals infected prior to vaccination displayed a sustained reduction in cumulative weight gain suggesting that early and prolonged hepatic damage or metabolic dysregulation can exert lasting effects. Importantly, within each experimental setting all groups were maintained under identical management, feeding, and environmental conditions. Therefore, the differences in weight gain observed between experimental groups are unlikely to be explained by ambient temperature alone. Interestingly, loss of weight gain started 48 wpi in both experimental settings, but only antiparasitic treatment in the second experimental setting attenuated these effects, suggesting that other factors can affect cattle productivity. Indeed, environmental and management-related factors, including climatic conditions and forage availability, are likely to modulate the productive impact of *F. hepatica* infection. Nutritional stress, pasture quality fluctuations, and thermal stress have been shown to exacerbate productivity losses associated with parasitic infections, even when parasite burdens are moderate, by increasing maintenance energy requirements and reducing feed efficiency (28, 29). Consequently, seasonal differences between experimental settings may have amplified the negative effects of fasciolosis on weight gain in one scenario, while more favorable environmental conditions may have facilitated partial recovery following antiparasitic treatment in the other, even though they were fed to reduce this variability as much as possible. In addition, the cumulative impact of parasite-induced hepatic damage on host metabolism represents a key contributing factor. *F. hepatica* infection disrupts critical metabolic pathways, including gluconeogenesis, lipid metabolism, and hepatic protein synthesis, leading to impaired nutrient utilization and reduced feed conversion efficiency, even in the absence of overt clinical disease (30). In animals experiencing prolonged infection and re-infection in natural settings where animals are continuously exposed to the parasite, these metabolic alterations may persist beyond parasite clearance, limiting the capacity of antiparasitic treatment to restore productive performance and potentially explaining the sustained reduction in weight gain observed in the first experimental setting.

Hematological findings were characterized by moderate leukocytosis and marked eosinophilia, independent of infection timing or treatment, reflecting sustained type 2 immune activation typical of helminth infections (14, 31). The absence of overt anemia or thrombocytopenia suggests that the infection burden achieved was sufficient to induce immunomodulation and hepatic injury without causing severe systemic hematological compromise, a profile consistent with experimental and natural infections by *F. hepatica* (28, 32). This pattern mirrors field observations in endemic regions, where subclinical fasciolosis frequently persists with minimal overt clinical signs but significant immunological and productive consequences (28, 30).

Biochemical analyses confirmed active liver involvement, with early elevations in AST indicative of hepatocellular damage during parasite migration, followed by increased GGT activity consistent with the development of chronic cholestasis (30). The more pronounced alterations observed in animals infected prior to vaccination likely reflect prolonged exposure to hepatic injury, supporting the notion that chronic liver dysfunction is a central component of fasciolosis-associated morbidity (28). The limited impact of TCBZ treatment on these biochemical parameters suggests that structural and functional hepatic alterations may persist beyond parasite elimination, as previously described in chronically infected cattle (12, 28). Furthermore, the reduction in the albumin-to-total protein ratio, particularly in animals infected earlier, indicates impaired hepatic synthetic capacity, a hallmark of chronic liver disease with potential systemic and immunological consequences (33). These results indicate that even when TCBZ reduces parasite burden, hepatic damage and immune alterations established during chronic infection may persist, suggesting that the effects of *F. hepatica* can outlast parasite elimination. These findings suggest that in endemic settings, where animals are frequently exposed to repeated or undefined infection events, factors such as reduced TCBZ efficacy and the stage of infection at which treatment is administered may influence the extent to which antiparasitic intervention restores optimal vaccine responsiveness, potentially through the persistence of parasite-driven immune regulation.

*F. hepatica* infection induced a parasite-specific IgG response detectable early after infection, followed by a gradual decline during the chronic phase, consistent with the humoral kinetics previously described in bovine fasciolosis (34). Antiparasitic treatment reduced the magnitude of this response, likely reflecting decreased antigenic stimulation following parasite elimination (12). This immunological profile aligns with the well-documented capacity of *F. hepatica* to induce robust humoral responses while simultaneously promoting regulatory immune pathways that limit excessive inflammation and favor chronic infection, including IL-10– and TGF-β–mediated mechanisms, expansion of regulatory T cells, and alternative macrophage activation (14, 17, 32, 35).

The humoral immune response to FMD vaccination against the A24/Cruzeiro strain followed kinetics consistent with those previously described, characterized by a rapid induction of IgG1 (36). Total antibody levels measured at 60 dpv exceeded the established protective threshold in all groups, indicating that *F. hepatica* infection, with or without TCBZ treatment, did not affect the magnitude of the anti-A24/Cruzeiro antibody response.

In contrast to antibody quantity, antibody quality was selectively affected by helminth infection. Although total anti-FMDV antibody levels and IgG1 titers were preserved, animals infected after vaccination exhibited a marked reduction in IgG1 avidity. As antibody avidity reflects the functional quality of the humoral response beyond antibody magnitude, this finding indicates a qualitative impairment of vaccine-induced immunity that would not be captured by conventional serological assessments alone. Similar results were described in naturally infected buffaloes (37).

In cattle, *F. hepatica* infection is considered acute during the migratory hepatic phase, occurring approximately between 2 and 8 weeks post-infection, whereas the chronic stage is established from 12–14 weeks post-infection onwards, when adult flukes reside in the bile ducts and egg shedding becomes detectable (38). This chronic phase is associated with sustained hepatic pathology and a pronounced immunoregulatory environment, which is particularly relevant for interference with vaccine-induced immune responses.

High-avidity antibodies are associated with enhanced virus-neutralizing capacity and improved cross-protection against antigenically diverse strains (4), and reduced IgG1 avidity has been linked to diminished cross-protective responses even when high antigen payload vaccines are used (7). Together, these observations suggest that *F. hepatica* infection interferes with post-vaccination affinity maturation processes, potentially compromising vaccine-induced protection under endemic conditions despite total antibody levels that are conventionally associated with protection.

The selective impairment of antibody avidity suggests that *F. hepatica* infection interferes with affinity maturation processes rather than with initial B cell activation or antibody production. Chronic helminth infections are known to skew immune responses toward Th2 and regulatory phenotypes, characterized by increased IL-4, IL-10, and TGF-β production and expansion of regulatory T cells (39). Such an immunological milieu can disrupt T follicular helper cell differentiation and germinal center dynamics, processes that are essential for the selection and survival of high-affinity B cell clones (40). The partial restoration of IgG1 avidity following antiparasitic treatment further supports the hypothesis that ongoing parasite-driven immunoregulation plays a causal role in shaping antibody quality.

In line with this mechanistic interpretation, the analysis of antigen-specific cellular responses revealed a consistent trend toward reduced IFN-γ production in animals infected after vaccination. Although these differences did not reach statistical significance, the magnitude and direction of the effect were reproducible across time points and were partially reversed by antiparasitic treatment. IFN-γ is a key cytokine associated with Th1 responses and has been implicated in supporting effective antiviral immunity and optimal germinal center reactions. Therefore, its reduction is consistent with the immunoregulatory environment induced by chronic helminth infection, characterized by Th2 polarization and increased regulatory cytokines. This shift may indirectly impair T follicular helper cell function and B cell selection processes, thereby contributing to the observed decrease in antibody avidity. The recovery of IFN-γ production following TCBZ treatment further supports the notion that ongoing parasite-driven immune modulation plays a role in shaping both cellular and humoral arms of the vaccine response.

Recent studies by Costa et al. (41, 42) demonstrate that *F. hepatica*-infected, vaccinated cattle retain total and subclass-specific anti-FMDV IgG levels, while exhibiting significant impairment in antibody avidity. Similar qualitative deficiencies have been reported in water buffaloes (*Bubalus bubalis*) naturally exposed to *F. hepatica* under field conditions (37), supporting the biological relevance of these findings beyond controlled experimental settings (41, 42). Together with the present findings, this evidence indicates that *F. hepatica*–driven immunoregulation does not primarily affect antibody production *per se* but rather interferes with the maturation and functional optimization of humoral responses, likely through altered germinal center activity and T follicular helper cell support.

Interestingly, animals infected prior to vaccination did not exhibit a significant reduction in IgG1 avidity, suggesting that the immunological context at the time of antigen exposure critically influences affinity maturation outcomes. Vaccination administered within an already established regulatory environment may allow compensatory mechanisms or alternative pathways of B cell selection, whereas disruption occurring during post-vaccination germinal center reactions may be more detrimental to antibody maturation. These observations underscore the importance of temporal interactions between helminth infection and vaccination in determining vaccine performance.

This study has, nevertheless, some limitations that should be acknowledged. First, the relatively small number of animals may have limited the statistical power to detect more subtle differences between groups. Second, although vaccine-induced humoral responses were comprehensively evaluated, no FMD virus challenge was performed; therefore, the impact of the observed changes in antibody quality on clinical protection was inferred rather than directly assessed. In addition, TCBZ treatment did not completely eliminate fecal egg shedding in all animals, which may have contributed to the persistence of parasite-induced immunomodulation. Although all experimental groups were maintained under identical environmental conditions, seasonal factors that influence cattle productivity cannot be completely excluded. Finally, these findings were obtained under controlled experimental conditions and should be further validated in naturally infected cattle exposed to repeated parasite challenge under field conditions.

In conclusion, these findings have important implications for FMD vaccination programs operating in regions where *F. hepatica* and other helminth infections are endemic. They indicate that chronic parasitic infection can compromise the functional quality of vaccine-induced immune responses without necessarily reducing antibody titers and cellular mediated-immune response, potentially creating gaps in protection that are not detected by conventional serological assessments. The partial restoration of immune quality following TCBZ treatment further supports the value of integrating parasite control measures with vaccination schedules. In addition, incorporating qualitative immunological readouts, such as antibody avidity, into vaccine evaluation frameworks may improve the assessment and effectiveness of vaccination programs across diverse livestock production systems.

## Data Availability

The raw data supporting the conclusions of this article will be made available by the authors, without undue reservation.
