# Supplementary material for: Timing of Fasciola hepatica infection selectively impairs antibody avidity after foot-and-mouth disease vaccination in cattle
Source: Front Immunol. 2026 Aug 11;17:1874141. doi: 10.3389/fimmu.2026.1874141 (PMC13504259; doi:10.3389/fimmu.2026.1874141)
Supplement: Supplementary file 1 [file DataSheet1.pdf]

Supplementary Figure 1

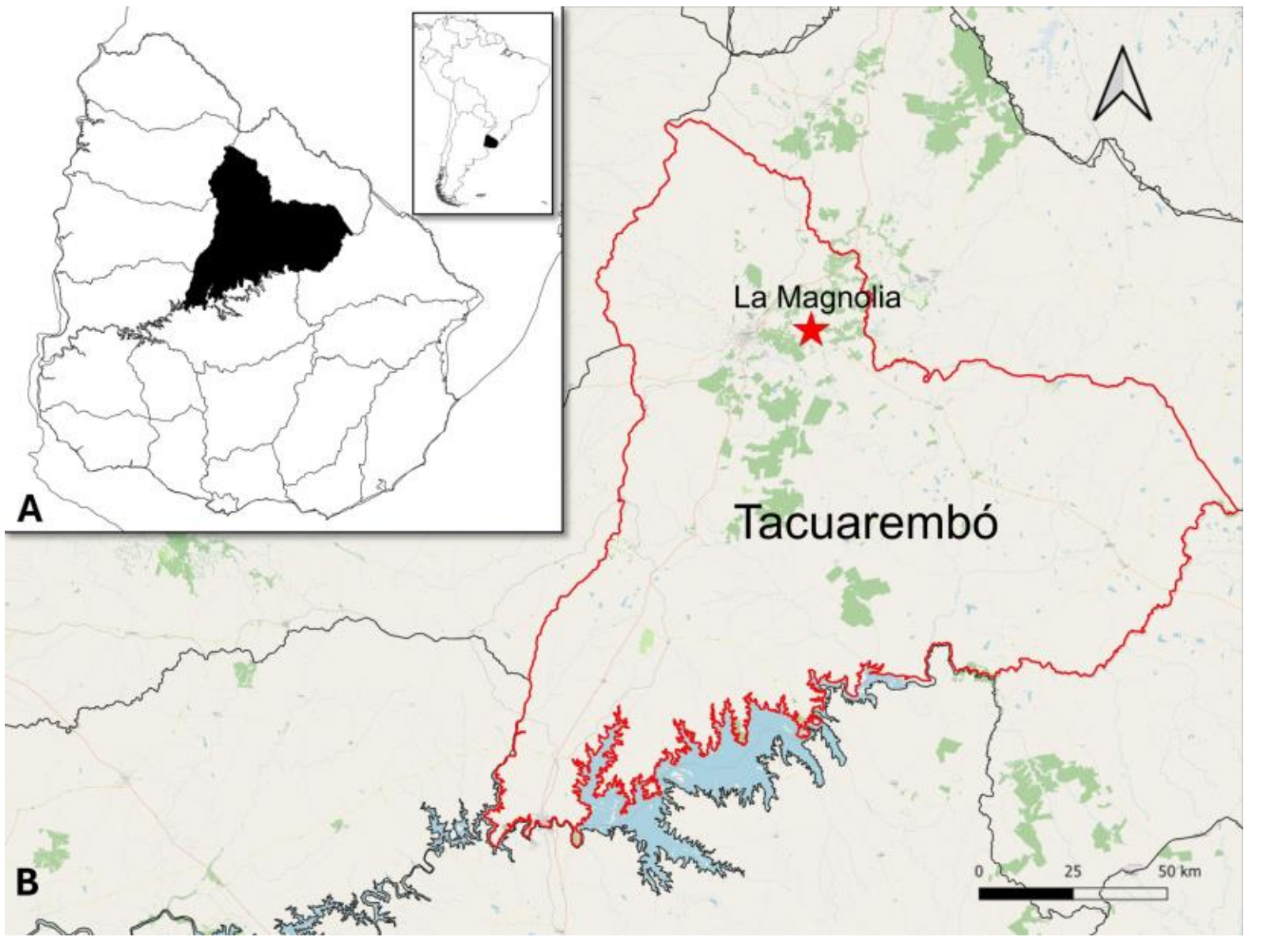

Supplementary Figure 1. A) Geographic location of the La Magnolia Experimental Station (INIA Tacuarembó, Uruguay), where the experimental trial was conducted. B) The station is located approximately 15 km east of Tacuarembó city (31°42′32″ S, 55°49′36″ W).

Supplementary Figure 2

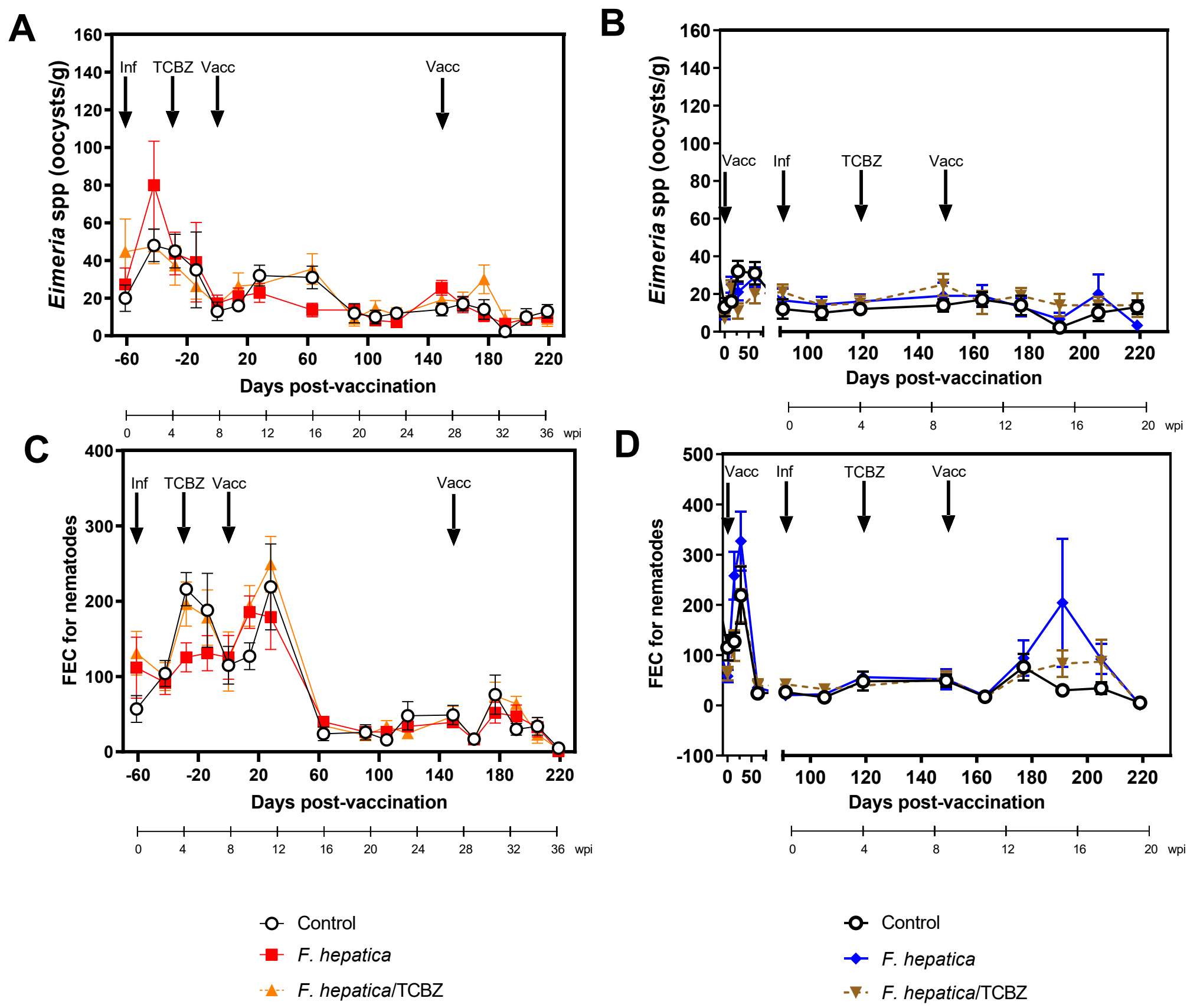

Supplementary Figure 1. Parasitic burden in infected animals. *Eimeria* spp (oocytes/g) were determined in animals infected prior (A) or after (B) vaccination. (B) Infection after vaccination. Fecal nematode Egg counts (FEC) per gram of feces were determined in animals infected prior (C) or after (D).

Supplementary Figure 3

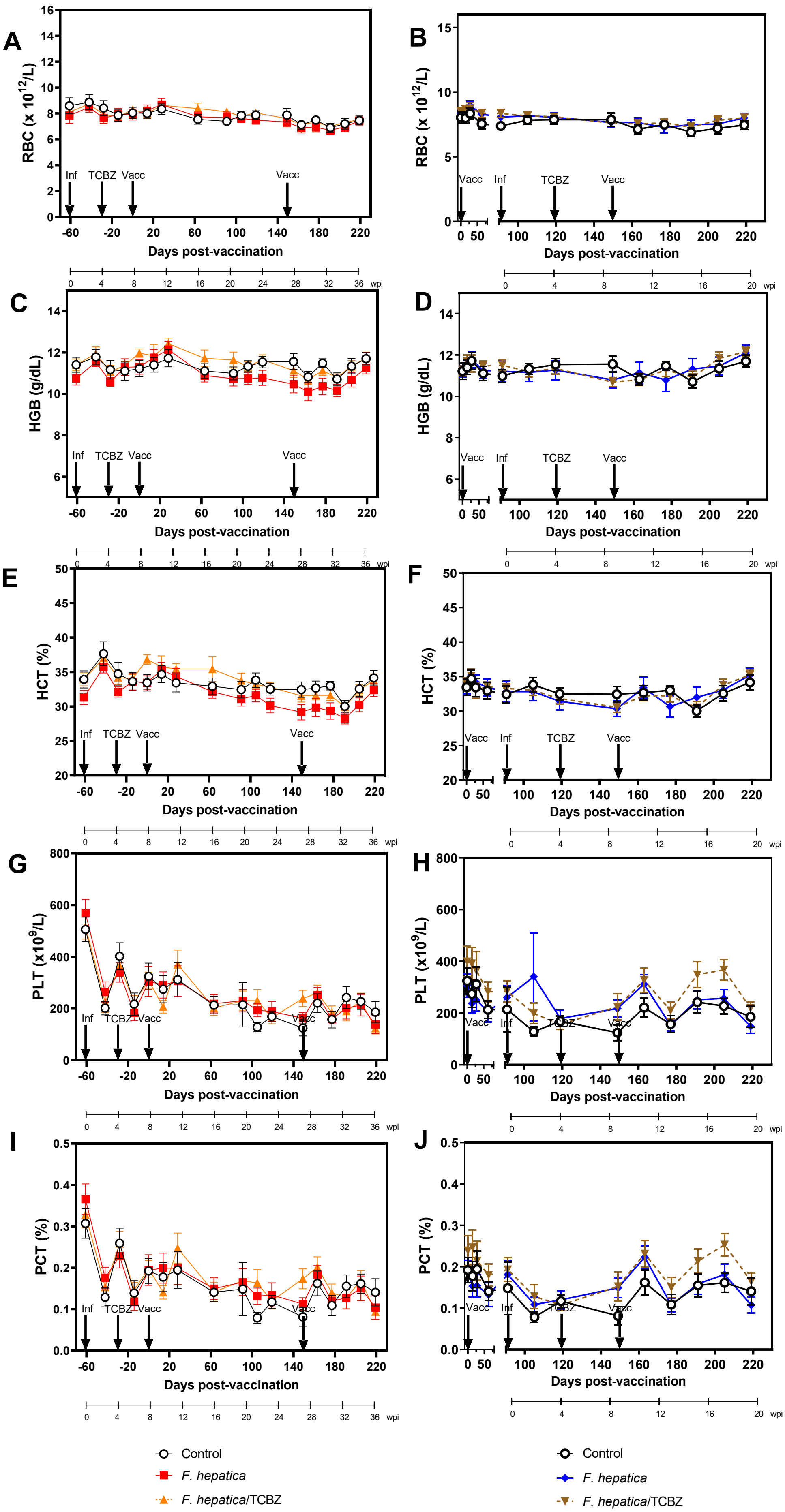

Supplementary Figure 2. A, B) Red blood cells (RBC). C, D) Hemoglobin B (HGB). E, F) Hematocrit (HCT). G, H) Platelets (PLT). I, J) Platelcrit (PCT). Animals infected prior (A, C,E, G, I) or after (B, D, F, H, J).
